# Comparative demography elucidates the longevity of parasitic and symbiotic relationships

**DOI:** 10.1098/rspb.2018.1032

**Published:** 2018-10-03

**Authors:** Luke B. B. Hecht, Peter C. Thompson, Benjamin M. Rosenthal

**Affiliations:** 1 Oak Ridge Institute for Science and Education, Oak Ridge, TN, USA; 2 US Department of Agriculture, Agricultural Research Service, 10300 Baltimore Avenue, Beltsville, MD 20705, USA

**Keywords:** demography, genomics, parasitism, symbiosis, domestication

## Abstract

Parasitic and symbiotic relationships govern vast nutrient and energy flows, yet controversy surrounds their longevity. Enduring relationships may engender parallel phylogenies among hosts and parasites, but so may ephemeral relationships when parasites colonize related hosts. An understanding of whether symbiont and host populations have grown and contracted in concert would be useful when considering the temporal durability of these relationships. Here, we devised methods to compare demographic histories derived from genomic data. We compared the historical growth of the agent of severe human malaria, *Plasmodium falciparum*, and its mosquito vector, *Anopheles gambiae*, to human and primate histories, thereby discerning long-term parallels and anthropogenic population explosions. The growth history of *Trichinella spiralis*, a zoonotic parasite disseminated by swine, proved regionally specific, paralleling distinctive growth histories for wild boar in Asia and Europe. Parallel histories were inferred for an anemone and its algal symbiont (*Exaiptasia pallida* and *Symbiodinium minutum*). Concerted growth in potatoes and the agent of potato blight (*Solanum tuberosum* and *Phytophthora infestans*) did not commence until the age of potato domestication. Through these examples, we illustrate the utility of comparative historical demography as a new exploratory tool by which to interrogate the origins and durability of myriad ecological relationships. To facilitate future use of this approach, we introduce a tool called C-PSMC to align and evaluate the similarity of demographic history curves.

## 1. Introduction

Symbiotic relationships, including parasitism, are cornerstones of functional ecology, governing vast nutrient and energy flows [1,2]. Yet their durability over evolutionary time scales remains controversial. Enduring relationships may give rise to parallel phylogenies among hosts and symbionts [3,4], but so may more ephemeral relationships when parasites disproportionately colonize related hosts [5]. In cases where ecologically crucial relationships have persisted for tens of thousands of generations, one would expect the populations of host and symbiont to have grown and contracted in concert.

The demographic history of a population summarizes changes in a multitude of factors, including its census size, sex ratio and gene flow with related populations, which together imprint on the genetic variability of the population. Population genetics measures demography in terms of effective population size (*N*_e_), defined as the minimum census size of an idealized population (a randomly mating population with an equal sex ratio, free from mutation, migration and differential reproductive success) required for it to harbour the observed level of genetic diversity [6].

The concept of effective population size is at the core of coalescent theory, which essentially models evolution in reverse through a population to derive statistical predictions regarding how long ago a pair of individuals or gene copies shared a common ancestor, or ‘coalesce’. The most fundamental insight of coalescent theory is that the average number of generations to coalescence is equal to twice the effective population size (2*N*_e_ generations).

Demographic history has typically been inferred by constructing gene trees based on multiple loci from many individuals in a population [7]. Just as species trees permit estimation of speciation rates over geological time, it is possible to recognize changes in coalescence rate from a gene tree based on the density of nodes (coalescence events) with respect to time [7,8]. Given that the average time to coalescence for a pair of alleles randomly drawn from a population is expected to be 2*N*_e_ generations, it may be inferred that a population was effectively smaller during periods when a disproportionate number of coalescences occurred. This makes intuitive sense; for example, during repopulation from a small set of founders immediately after a bottleneck, most alleles must have coexisted in one or more of the founders. This is the basis for demographic history inference using population genetics.

The pairwise sequentially Markovian coalescent (PSMC) model [9] broke new ground through the insight that by applying the coalescent with recombination [10] to a diploid genome, the mosaic ancestry of different segments of the genome permit detailed demographic history reconstruction on the basis of a single individual. It does this based on the frequency distribution of loci featuring given proportions of heterozygous sites, relative to expectations for a constant population size under the coalescent where heterozygosity of the entire genome defines the constant effective population size. The end result of PSMC analyses is a demographic history curve that depicts effective population size moving backwards through time on a log scale.

Many studies using PSMC and similar methods have attempted to correlate features of demographic history with environmental events, such as the Last Glacial Maximum (LGM). For example, Groenen *et al*. [11] used PSMC to show that the effective population size of wild boar grew after boar from Asia first colonized Europe, relatively free from competition. Subsequently, both European and Asian populations experienced bottlenecks around the time of the LGM, although the bottleneck was stronger in Europe, probably due to more extensive glaciation.

On the other hand, little attention has been paid to correlations between the demographic histories of different species that have close ecological ties. This is probably because most studies focus on only one or a handful of species with a common ancestor. One exception is the work of Zhou *et al*. [12], which found that the demographic history of leaf-eating snub-nosed monkeys (*Rhinopithecus roxellana*) correlates much more closely with that of leaf-eating pandas than of its congeners, suggesting diet as a historically primary factor in how the population fared. If this interpretation is correct, one might suppose that the demographic histories of the plants they eat are also correlated. For their analysis, Zhou *et al*. used published estimates of mutation rate and generation time for each species. However, such estimates often involve considerable uncertainty and variance between methods, and generation time could vary between populations and time periods. This is an important consideration when comparing demographic history curves, because slight differences in scaling can severely bias quantitative evaluations of coordinated changes in abundance, especially when the demographic history is relatively complex (i.e. reflects multiple episodes of population growth and decline).

Here, we devised methods to compare and optimally align demographic histories derived from genomic data [9], and applied them to a broad array of parasitic and symbiotic relationships in order to gauge their power to evaluate the origins and temporal durability of such biological dependencies, emphasizing the utility and ease of this novel approach for exploratory analysis and hypothesis generation. We used these methods to compare the historical growth of the agent of severe human malaria, *Plasmodium falciparum*, with human and primate histories [13,14] and with that of their mosquito vector *Anopheles gambiae* [15], thereby discerning long-term parallels and anthropogenic population explosions [16,17]. We examined the evidence for geographically specific demographic parallels in swine [11] and the agent of trichinellosis [18]. We investigated whether a marine symbiosis between an anemone [19] and its photosynthetic dinoflagellate [20] have undergone concerted population fluctuation in spite of recurrent, climate-driven bleaching events. Finally, we determined whether the heterokont agent of potato blight [21] underwent common population dynamics with potatoes [22] themselves prior to their domestication. The comparison method has been packaged into a software tool called Comparative PSMC (C-PSMC), which optimally aligns PSMC files in order to score their similarity and estimate relative evolutionary rates.

## 2. Methods

In order to examine the demographic history of individual species, we determined the extent and distribution of heterozygous positions called from high-quality genome assemblies. Each demographic history was then reconstructed using the PSMC model, a procedure that uses the distribution of heterozygous sites in a diploid genome to estimate the size of the diploid individual's ancestral population over time [9]. The output of PSMC must be scaled into absolute terms of time and population size using estimates of mutation rate and generation time. To synchronize the resulting demographic history plots, we devised a quantitative curve-fitting procedure to estimate the relative evolutionary rate of organismal pairs, within a species-specific plausible range of mutation rate and generation time. We then assessed whether and when ancestral populations grew or contracted in concert. To explore the power of these methods, we tested whether natural species pairs (e.g. malaria and humans) matched each other significantly better than arbitrary pairs (e.g. malaria and wild boars). We further established the temporal specificity of this approach by verifying that synchronized demographic plots fit each other significantly better than chronologically disordered comparisons.

Here, we describe in detail our means of acquiring sequence reads, assembling them into scaffolds, calling heterozygous positions and analysing the distribution of heterozygous positions using the PSMC model [9]. We then describe methods for assessing the goodness of fit between pairs of demographic plots and determining the extent to which synchronized, natural pairs of plots better align than do plots of arbitrary species pairs or natural pairs lacking proper temporal order.

### (a) Sequencing

Short-read whole-genome sequencing was used to produce novel genome assemblies of *Trichinella spiralis* isolates from Asia and ‘Western’ samples from Maryland and New Hampshire, USA. Isolates were collected from carcasses of animals determined to be infected post-mortem. Western samples were collected for molecular epidemiology purposes by the US Department of Agriculture. Genomic DNA from Asian isolates was graciously provided by Dr Ming-Yuan Liu, Jilin University, Changchun, China. Genomic DNA was extracted using Promega DNA-IQ magnetic bead technology with the Tissue and Hair Extraction Kit Protocol according to manufacturer instructions. Extracted DNA was fragmented using Illumina's NexteraXT DNA Library Prep Kit according to manufacturer instructions. After quantifying libraries, paired-end 300-base pair sequencing reads were generated on an Illumina MiSeq using the Illumina MiSeq Reagent Kit v3 chemistry. Each read was trimmed such that no base would have a higher than 5% chance of an error, and only two bases with quality scores less than Q20 would pass into the final read. Following quality control, reads longer than 50 bases were mapped to reference scaffolds using the Geneious Assembler with default settings in Geneious v. 10.2.3 [23] (Biomatters Ltd, New Zealand).

### (b) Previously published genomes

Genomes from species of interest were obtained from the public databases hosted by the National Center for Biotechnology Information at the National Institutes of Health. For each species, the chromosome or scaffold sequences were downloaded for use as reference sequences. Corresponding sequence read archives (SRA files) from individual samples were downloaded and subjected to quality control measures described above. Individual reads were mapped to the chromosome/scaffold sequences using Geneious Assembler in Geneious v. 10.2.3. Accession numbers for scaffolds/chromosomes and SRA (files) are listed in electronic supplementary material, table S2. For *Pl. falciparum*, which was sequenced in its haploid form, pseudodiploids were generated by mapping reads from two separate isolates from a geographical region onto a single reference sequence.

### (c) Variant calling and determination of consensus sequences

Variant-calling was performed on all assemblies using Geneious v. 10.2.3. Consensus base calls were derived from the 75% of reads with the highest representation at any particular locus. If sequencing depth at any base was less than 5×, that base was considered indeterminate (*N*) and, as such, ignored by PSMC analyses. The consensus for each chromosome was exported in FASTA format for use in subsequent analyses using IUPAC nomenclature to denote heterozygotes.

### (d) The pairwise sequentially Markovian coalescent

Our approach examines whether, to what extent and when pairs of PSMC plots correspond. The estimates of PSMC derive from the frequency of genomic loci, inferred by the model of recombination, that is defined by a given level of heterozygosity. The underlying coalescent model considers the stochastic processes of mutation accumulation and the persistence or loss of ancestral alleles. It further considers the effects of recombination in breaking down blocks of co-inherited sequence. Actual distributions of heterozygosity are considered against the frequency distribution of heterozygous loci expected for genomes descended from populations of constant size. Disproportionately frequent occurrences of loci defined by a given level of heterozygosity are taken as evidence that the ancestral population waned, while the date of such a bottleneck is estimated from the temporal interval over which that level of heterozygosity would have been expected to accumulate. Similar intuition governs this model's estimates of episodes of population growth (during which fewer-than-expected coalescence events would have occurred). The end result of any PSMC analysis is a plot of effective population size through time, limited by the detection of adequate numbers of coalescent events in the recent past and the ability of the algorithm to identify loci that coalesce in the remote past, characterized, respectively, by the lowest and highest ranges of heterozygote density represented in the genome.

**Figure 1.**
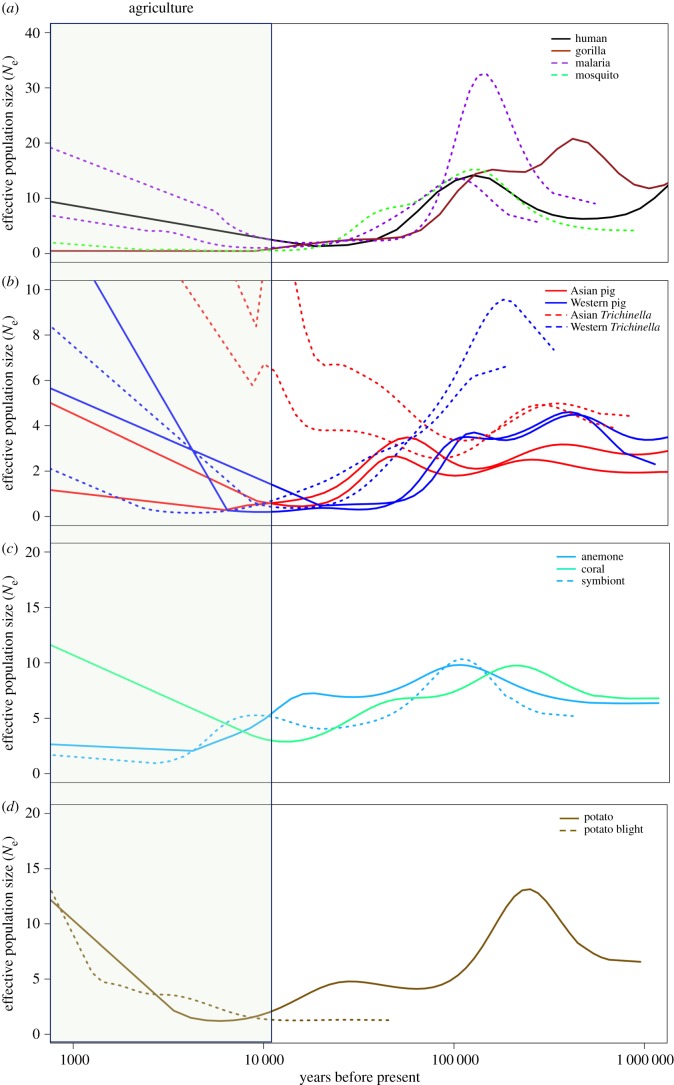
PSMC plots comparing the demographic histories of hosts and their putative parasites/symbionts. The vertical axis is scaled to 10^4^. The time period associated with human agriculture (most recent 12 000 years) is shaded. Solid lines represent hosts, whereas dotted lines represent their parasites or symbionts. (*a*) *A. gambiae* mosquito and two *Pl. falciparum* parasite populations appear to have grown and contracted in sync with human, but not gorilla, populations over the last 500 000 years. Demography estimated from each of two pseudo-diploid malaria genomes illustrated (see electronic supplementary material, methods). (*b*) Both Chinese *T. spiralis* isolates (red dashed lines) mirror the demographic history of sympatric members of their host species (solid red lines), while North American *T. spiralis* (blue dashed lines) was more similar to wild boar and pig populations from Europe (solid blue lines). (*c*) The algal symbiont *S. minutum* has tracked the demography of its known host, the anemone *E. pallida*, rather than the coral *Acropora digitifera*, at least since the LGM (approx. 20 ka). (*d*) *Phytophthora infestans*, the agent of potato blight, only shows concerted growth or decline with potatoes since domestication.

PSMC analysis was performed on each genome assembly individually. In order to obtain robust population size estimates in the geologically recent past, the number of linked time parameters in the ‘-p’ input was varied such that the most recent time interval would encompass a minimum of 100 inferred coalescent events. This threshold is 10 times more stringent than recommended in the PSMC documentation as a minimum to avoid overfitting. The output of PSMC is initially scaled relative to the overall heterozygosity of the genome being analysed, so it must be scaled into terms of absolute time and effective population size by assuming a mutation rate and generation time. We assumed an approximate mutation rate of 2.5 × 10^−8^ per site per generation for all species except the mosquito, *A. gambiae*, which is thought to have a rate closer to that of *Drosophila*, at 1.1 × 10^−9^ per site per generation [24]. Generation time was allowed to vary within limits, and then evaluated after aligning pairs of PSMC curves (figure 1) as described below.

### (e) Fitting PSMC curves

Each parasite curve was fitted to all possible host curves, seeking to maximize intervals of shared population growth or decline by minimizing differences in slope. As the rate of growth or contraction (steepness of curve slope) might differ between host and parasite, we found optimal fit based on a simple ternary system of negative growth, stasis or positive growth (assigned values of −1, 0 and 1, respectively). For each species pair considered, the host PSMC demographic curve was held constant while the relative generation time was allowed to vary for each symbiont from 0.001 to 2.000 (i.e. one-thousandth to twice the generation time of the host) in 0.001 increments, assuming a constant generational mutation rate. This varied the temporal scaling of the PSMC curve without changing its shape. For each incremental change in generation time, the slope sign (−1, 0 or 1) was determined at successive 1000-year time-points and the average difference in slope between putative species pairs was calculated. The average slope difference was recorded as a curve-fitting metric, where values of 0 indicate a perfect match and 2 an inverse correlation. The minimum average slope difference across the range of generation times was taken as the optimal fit, and the relative generation time that optimized fit was noted for comparison with known life history of the organisms in question (electronic supplementary material, table S3). This approach accounted for a degree of uncertainty in the PSMC estimates of effective population size at each point in time and helped to avoid spurious fits based on the overlap of only a small proportion of the two PSMC curves. The final optimal fit was visualized as a superimposed PSMC plot with all relevant hosts and parasites (figure 1). Software for implementing this approach, C-PSMC, is described and available for download at https://github.com/lbbhecht/C-PSMC.

The correspondence between the demographic histories inferred for extant host–symbiont relationships was compared, using this curve-fit metric, with alternative ‘plausible’ pairs. After completing the curve-fitting for all plausible hosts within a system (e.g. malaria with humans or gorillas), the host with the smallest average slope difference was deemed the best fit, and thus the ‘natural host’ responsible for controlling the growth or decline of the parasite of interest over the timeframe encompassed by the PSMC plots. This designation was used for further validation analyses described below.

Finally, the relative generation times for each symbiont species could be translated into absolute units of time (years or months) using the combination of mutation rate and generation time appropriate for the best-fit host. We note, with interest, that this same method could be used to instead estimate relative mutation rates for any pair of species for which generation times are known *a priori*.

### (f) Testing curve fit

Any curve-fitting procedure might be capable of fixating on spurious parallelism, even among species pairs sharing no true history. We therefore sought to verify that observed parallels were more than chance occurrences.

**Figure 2.**
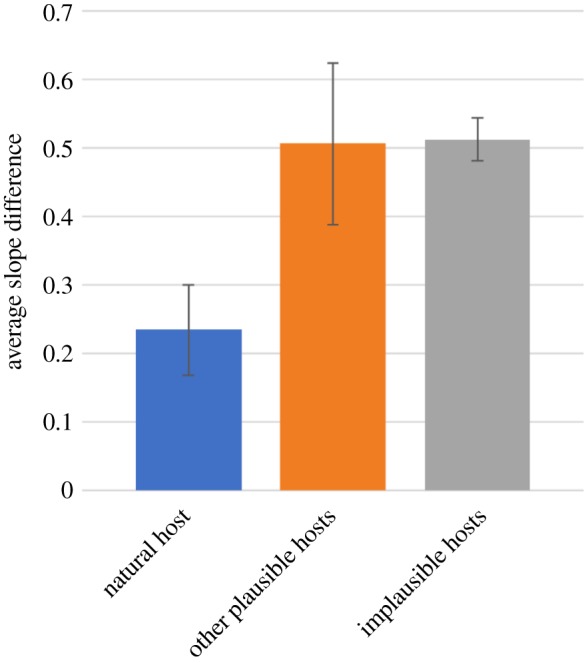
Comparison of minimum average slope difference for the natural host (e.g. *Pl. falciparum* × human; *n* = 13) versus plausible (e.g. *Pl. falciparum* × gorilla; *n* = 12) and implausible (e.g. *S. minutum* × human; *n* = 56) alternative host–symbiont combinations. Error bars represent the 95% confidence interval of all comparisons. (Online version in colour.)

One means to do so entailed comparing the fit of natural species pairs (defined by extant parasitic or symbiotic relationships) to plausible relationships (defined by historic associations of hosts and parasites) to ‘implausible’ species pairs. For example, there would be no strictly biological reason to expect parallel demographic histories for *Pl. falciparum* with pigs, *T. spiralis* with potato, *Phytophthora infestans* with gorillas, or this species of *Symbiodinium* (*S. minutum*) with a species of coral (*Acropora digitifera*) with which it has not been shown to establish a symbiosis. In so doing, we sought to test whether our curve-fitting approach was so flexible as to identify parallels where none should have been expected, *a priori*. Thus, each parasite/symbiont PSMC plot was fitted, respectively, to all host species in the entire study as described above. At the optimal fit for these implausible hosts, the average slope difference was calculated (electronic supplementary material, figure S1). The average minimal slope difference of parasites to all natural hosts, other plausible hosts and implausible hosts was calculated along with 95% confidence intervals (figure 2).

**Figure 3.**
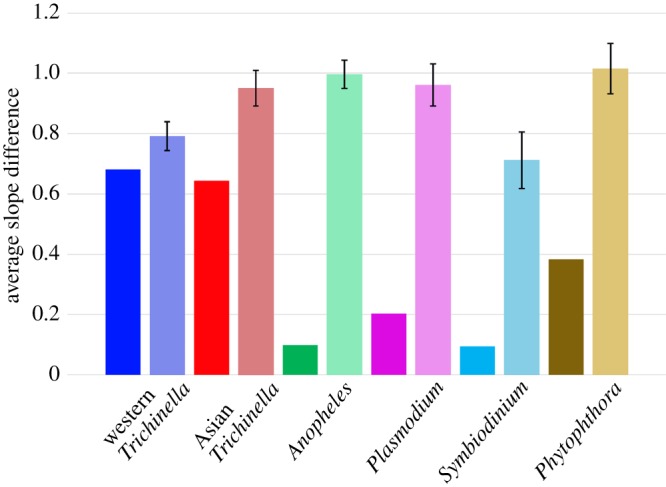
Comparisons of average slope difference between unmodified curves (solid colour) and curves that have been randomized with respect to time (faded). Symbiont plots were aligned to their purported host plots (Western *T. spiralis* versus Western *S. scrofa;* Asian *T. spiralis* versus Asian *S. scrofa*; *Pl. falciparum* versus human; *A. gambiae* versus human; *S. minutum* versus *Exaiptasia pallida*; *Ph. infestans* versus potato) and the average difference in slope direction was calculated. The 99.9% confidence interval is shown in association with the randomized curves, based on the variance among randomization replicates (*n* = 5). Randomizing slopes with respect to time significantly worsened the match for every ecologically relevant pairing of species. (Online version in colour.)

In another attempt to evaluate the extent to which shared histories probably explained parallel demographic plots, we determined whether a parasite's population growth more closely mirrored contemporaneous growth in its host than it mirrored growth in its host at arbitrary times. For example, if the entire reconstructed history of a host was dominated by growth, every time-point would have the same slope and the order would be irrelevant. To carry out this test, we randomized the slopes of each sample with respect to time, separately, for each set of samples. Correctly ordered slopes from one species were then compared to non-chronological ones from the other. A two-tailed *t*-test was used to evaluate whether the mean slope differential for contemporaneous plots was less than when one plot's temporal order was randomized with respect to the other, based on five independent randomizations (figure 3). In general, the more complex the curve (i.e. the more local maxima/minima), the more power we should have to determine goodness of fit.

## 3. Results

### (a) Malaria system

We found that *Pl. falciparum* and *A. gambiae* populations grew and shrank in concert with human demography over an approximately 500 000 year interval, and not with other great apes (figure 1*a*; electronic supplementary material). The minimum slope difference between *Pl. falciparum* or *A. gambiae* and human demographic curves averaged 0.07, with a generation time of 0.48 years and 0.057 years, respectively. Populations grew steadily, tripling between 500 and 200 ka, then declined over the following 170 kyr to about 20% their maximum, and stabilized for approximately 20 kyr. Populations then began to grow 3–11 ka.

### (b) *Trichinella* system

Demographic history was highly reproducible between two sequenced isolates of *T. spiralis* from Asia and two from the West, as well as their porcine hosts (figure 1*b*). The minimum average slope difference between Asian *T. spiralis* and Asian suids (0.30) occurred at a parasite generation time of 1.1 years, while differing from European pig or boar curves by an average of 0.48 using growth/decline/stasis metrics. On the other hand, Western *T. spiralis* curves had lower minimum slope differences with European suids (mean = 0.30) than with Asian pig or boar curves (mean = 0.41). Populations from both continents peaked approximately 300 ka, followed by a cycle of decline and growth. However, they differed markedly over much of the last 100 kyr, as Western *T. spiralis* and pig populations began a terminal decline approximately 100 ka, while Asian pig populations began a similar decline only approximately 50 ka. At the same time, slowed yet continual growth is observed for Asian *T. spiralis*. In the most recent 10 000 years, the populations of both continents and species experienced moderate to extreme growth.

### (c) *Symbiodinium* system

Prior to the LGM, similar patterns were observed for ancestors of an anemone (*Exaiptasia pallida*) and its photosynthetic algal symbiont (*S. minutum*), as well as *A. digitifera*, a coral (figure 1*c*). The slope of the *Symbiodinium* demographic curve differed from its natural host*, Aiptasia*, by only 0.10 at a generation time of 0.52 years, while the slope difference with the unrelated coral was larger (0.17), with a longer generation time (0.97). Each of their demographic histories reveal relative maxima at approximately 20–40 ka and approximately 100–200 ka. However, following the LGM, the coral population experienced major net growth over the last 10 000 years while the anemone and alga experienced decline and stasis.

### (d) Potato blight system

There was little correspondence between the effective population sizes of the ancestors of potato and the agent of potato blight (*Ph. infestans*) prior to approximately 10 ka, but after that time they both grew rapidly (figure 1*d*). The minimum average slope difference between *Ph. infestans* and potatoes was 0.38, higher than the minimal slope difference with three other unnatural hosts: European boar (0.06), Chinese pig (0.27) and anemone (0.33). The reconstructed history of *Ph. infestans* was relatively brief and static, extending back only to approximately 50 ka. The potato genome, on the other hand, evidenced a deep demographic history with relative peaks at approximately 20 ka and approximately 200 ka, as seen in the *Symbiodinium* system.

### (e) Method validation

To validate the specificity of our curve-fitting method, we optimally aligned the PSMC curve of every symbiont to that of every host, respectively, and compared the goodness of fit between each pairwise alignment. Overall, natural pairs exhibited around half the average slope difference (i.e. twice as good a fit) of alternative pairings, while no difference was observed between plausible and implausible alternative pairings (figure 2). The results for individual host–symbiont pairings are shown in electronic supplementary material, figure S1.

We also sought to determine whether there was, fundamentally, enough information in the simple slope direction of the curves to distinguish a true historical fit from a chance occurrence. To do this, we randomized the temporal order of symbiont slopes relative to those of their natural host and compared the average slope difference to that calculated when the host and symbiont curves were optimally aligned in the correct temporal order (figure 3). Overall, randomization increased the average slope difference by a mean of 158% (*p* = 0.0009).

## 4. Discussion

### (a) Method validation

We demonstrated temporal and biological specificity in the demographic correspondence of host and parasite populations. Temporally ordered histories corresponded to a significantly greater degree than did temporally disordered comparisons, and natural host–symbiont pairs matched twice as well, on average, than did apocryphal pairings. In all cases, the true host was preferred from the arena of plausible hosts (e.g. European boar > Asian boar for Western *T. spiralis*), although in some cases—namely, ones where one or both the PSMC curves were relatively lacking in complexity—an apocryphal match scored well (e.g. European boar with *Ph. infestans*). The patterns evident in each system warrant further interpretation.

### (b) Malaria system

Falciparum malaria and its mosquito vector grew and contracted in concert with humans (and certain populations of chimpanzees, but not with gorillas) prior to 100 ka. Notably, these parasites and vectors paralleled the explosive growth unique (among primates) to human beings since the Neolithic, complementing a recent comprehensive survey of anopheline populations [16]. Our curve-fitting procedure yielded estimates of generation time closely approximating prior empirical estimates. Assuming 25-year generations for primates, we estimated for *Pl. falciparum* and *A. gambiae* 2.08 and 17.5 generations per year, respectively, even without any constraints on the range of biologically plausible values (previously reported as 2 and 10–24 generations per year, respectively [17,24,25]). Our findings support prior conclusions that forest clearing and agricultural settlements engendered growth in anopheline populations and intensified their role as vectors of malaria [26].

It has been proposed that *Pl. falciparum* shifted from gorillas to humans between 60 000 and 130 000 years ago [13]. Chimpanzees had previously been considered the penultimate host for the ancestors of falciparum malaria until more closely related parasites were discovered in gorillas*.* Because ancestors of chimpanzees may have served as earlier hosts to ancestors to falciparum malaria, we considered the demographic history of *Pl. falciparum* in relation to that published by Prado-Martinez *et al*. [14] for various populations of chimpanzees (*P. troglodytes ellioti*, *P.t. schweinfurthii*, *P.t. troglodytes*, *P.t. verus*). While all Old World primates (including *H. sapiens*) share certain features of demographic history, the histories of various sub-populations of chimpanzees notably differ (electronic supplementary material, figure S4). The demographic history of *Pl. falciparum* tracks human demography best, especially during the most recent historical interval, when human populations grew exponentially while other great apes did not. However, of the considered chimpanzee populations, *P.t. schweinfurthii* was the most consistent with human and malaria demography in the range of 80 ka to 1 Ma.

### (c) *Trichinella spiralis* system

The growth history of *T. spiralis*, a zoonotic parasite disseminated by swine domestication [27,28], proved regionally specific, paralleling distinctive growth histories for wild boar in Asia and Europe [11]. Asian *T. spiralis* specifically grew and contracted with sympatric wild boar, growing between 800 and 400 ka, declining and then growing 100–40 ka; populations of boar in Europe, by contrast, peaked approximately 100 ka and declined until approximately 10 ka. In North America, *T. spiralis* are so lacking in heterozygosity (presumably owing to a severe population bottleneck) that PSMC cannot be used to reliably estimate demographic history before 200 ka (figure 1*b*). In spite of this truncated and relatively simple demographic reconstruction, temporally ordered comparisons of Western *T. spiralis* and European wild boar match significantly better than do asynchronous comparisons (figure 3). Highly reproducible demographies from 100 to 10 ka substantiate explosive growth of *T. spiralis*, in each region, during the last 20 kyr (figure 1 and electronic supplementary material). Transmission evidently intensified after the advent of agriculture, probably among abundant (but inbred) domesticated swine and rats.

### (d) *Symbiodinium* system

In spite of climate-driven bleaching events that might have rendered ephemeral the symbiotic relationship between an anemone and its photosynthetic symbiont (*E. pallida* [19] and *S. minutum* [20]), strikingly similar and temporally specific demographic histories were recovered (figure 1*c*; electronic supplementary material). A long-term demographic association between *E. pallida* and *S. minutum* might not have been expected owing to shuffling of symbionts during periods of environmental stress [29,30]. Following heat stress, thermally tolerant symbionts have been shown to become numerically dominant in communities of cnidarians [31], albeit with possible energetic costs [32]. Although such associations might therefore prove ephemeral, the population of *S. minutum* tracked that of *E. pallida* remarkably closely (figure 1*c*; electronic supplementary material, figure S8). The demographic histories of all three species were similar until approximately 10–20 ka, following the LGM, when the coral population began a period of extreme growth while the anemone and symbiont populations began to decline. Their shared history in the Red Sea may partially explain the evidently shared demographic histories of the anemone and its symbiont (in contrast to that of the coral, obtained from Okinawa in the East China Sea).

Considering the foundational role of corals in creating habitat for anemones and their symbionts, it is not surprising that the demographic histories of all three species are very similar in overall shape. Indeed, the histories of *Aiptasia* and *A. digitifera* can be nearly superimposed by assuming a generation time ratio of 1 : 2 (data not shown). Although we have assumed a default generation time of 1 year for *A. digitifera* and *Aiptasia*, given published data, *A. digitifera*'s generation time could be as long as its age of maturity (3–8 years [33]), which would imply a similar generation time for *S. minutum* and one of 6–16 years for *Aiptasia*. That would be an implausibly long generation time for a dinoflagellate, but this relative scaling of demographic history could also be accounted for by a slower per-generation mutation rate. Indeed, the typical generational mutation rate of other haploid eukaryotic phytoplankton (approx. 5 × 10^−10^ per site per year) is around 50 times slower than the mammalian one we have assumed by default (2.5 × 10^−8^) [34]. This would imply an *S. minutum* generation time of approximately three to eight weeks, rather than 3–8 years. Without better estimates of mutation rates for each of the organisms involved, the inferred generation times should be treated as guidelines. Nevertheless, the shape of the PSMC curves support similar demographics among all three species prior to 20 ka.

### (e) Potato system

Infections of potatoes (*Solanum tuberosum*) with the potato blight *Ph. infestans* caused one of the greatest human disasters in agricultural history, resulting in the death or emigration of 20% of the Irish in the mid-1800s. Potatoes originated in the Andes and may have been domesticated twice. Biogeographic data suggest the central Mexican highlands as the ancestral home of *Ph. infestans,* achieving global reach only after the potato domestication in the Neolithic and the globalization of agriculture beginning 500 years ago.

Concerted growth in potatoes and the agent of potato blight (*Solanum tuberosum* [22] and *Ph. infestans* [21]) did not commence until the age of potato domestication, helping date the acquisition of this historically consequential plant pathogen. Our results show that prior to potato domestication and cultivation, potatoes and the agent of potato blight shared no obvious episodes of concerted population growth or contraction. Lacking such compelling information, and with a relatively short and simple reconstructed history of *Ph. infestans* to overlap, our curve-fitting algorithm easily identified a clear preferred relative generation time for the two at approximately 0.14 *Ph. infestans* generations per potato generation (which was assumed to be 1 year; electronic supplementary material, figure S9). Adopting this temporal scalar resulted in a fit that was as poor as the best fit between apocryphal species pairs (such as wild boar), even with relatively tight constraints on plausible generation times for the pathogen (less than 3 months [35]). With few options, the best-fit curves register by reference only to the recent (post-domestication) interval of population growth in potatoes. This is preceded by approximately 20 000 years of decline in potatoes and relative stasis in *Ph. infestans* (figure 1*d*; electronic supplementary material, figure S10). These findings are consistent with a close affinity of *Ph. infestans* with potatoes only since their domestication.

*Phytophthora infestans* appears restricted to tuber-forming species of *Solanum* and does not easily cross with other species of *Phytophthora*. The closest known relatives to *Ph. infestans* (*P. andina, P. ipomoea, P. mirabilis* and *P. phaseoli*) are also pathogens of plants native to the Neotropical highlands. However, other closely related congeners (*P. iranica, P. clandestina, P. tentacultate*) afflict diverse plant types (aubergine, clover, chrysanthemum) in diverse regions (Asia, Europe). An even greater diversity of host types and biogeographic regions characterizes more inclusive groups of *Phytophthora,* including species infecting orchids native to Indonesia, lilacs in the Balkans, rhododendron native to the Himalayas, raspberry native to Northern Europe and Asia, and Douglas fir in western North America. This broader evidence for host switching provides valuable context to our results, suggesting parallel demography only during the most recent past.

These cases illustrate the utility of comparative historical demography as a powerful new means by which to interrogate the history of myriad ecological relationships, enriching our understanding of their origins and durability.

Our approach builds on prior achievements extracting demographic information from heterozygosity patterns. Genomic regions whose evolutionary histories have been separated by recombination harbour distinctive amounts of heterozygosity, enabling inferences about the effective population size assuming a model of time-to-coalescence. In principle, this approach can be used to examine the degree of correspondence among any pair of high-quality genome assemblies in which heterozygous positions have been identified, provided that genome scaffolds are of sufficient length to capture the requisite recombination blocks. Here, we reexamined published inferences derived from PSMC (in the cases of wild boars and primates), performed our own genome sequencing and analysis (in the case of *T. spiralis*), and mapped reads to existing assemblies that had not been previously subjected to PSMC analyses (anemone and its dinoflagellate symbiont, falciparum malaria, *Anopheles* mosquitos, potato, *Ph. infestans*, and some wild boar and domestic pigs).

The merit of this approach lies in generating new hypotheses from exploratory analyses, requiring a minimum of samples and time. We have chosen to illustrate this using PSMC, which is now treated as one of the default analyses to apply to any newly sequenced diploid genome. However, given a much greater number of sample genomes, other methods could be applied, such as the recently described SMC++ [36], providing much greater confidence in the demographic history reconstructions while remaining compatible with the tools and approach we have developed here for comparative historical demography.

Pearson's correlation coefficient was previously used to evaluate the goodness of fit between demographic curves [12], given presumed mutation rate and generation time scalars. We found this approach notably sensitive to the magnitude and relative timing of changes in effective population size; such fluctuations may be influenced by intrinsic biological factors much more than concerted, ecologically driven population changes. Moreover, published estimates of mutation rate and generation time for a given species often vary, and even generation time may vary among populations and over time. Domestication, for example, imposes new imperatives on the reproductive interval. This is an important consideration when comparing demographic history curves because even slight differences in scaling can bias estimates of the fit between two curves, especially when the demographic history is relatively complex (i.e. reflects multiple episodes of population growth and decline).

The demographic events reconstructed by PSMC may include those affecting not only extant species but also their corresponding ancestral species. This enables one to ask whether even the remote ancestors of a parasite may have parasitized the ancestors of its extant hosts. Joint analysis of the coalescent proves capable of discerning whether, and for approximately how long, a pair of organismal populations have grown and contracted in concert.

Gene flow between genetically diverging incipient species could bias estimates of effective population size upward based on anomalously high genetic diversity during that time. Like all coalescent analyses, PSMC-based inferences of demographic history are subject to potential biases introduced by population structure or selection, which can (respectively) inflate or deflate estimates of population size [37]. However, where population structure exists in a host species, its effects may be mirrored in the genetic diversity of a co-evolving symbiont [38]. Given inevitable uncertainty in the values of mutation rate and generation time that go into scaling the output of PSMC into years and effective population size, as well as the small sample size the model is designed for (a single diploid genome), the absolute numbers should not be taken too literally. Thus, our approach focuses only on the direction of change in population size.

When introducing PSMC by application to human genomes, Li & Durbin [9] discounted the most recent 20 000 years of reconstructed history as unreliable, and most subsequent analyses have followed suit, leaving unexamined the ascendance of countless species (our domesticates, weeds, pests and pathogens) and the demise of countless others (by hunting, habitat destruction, pollution and climate forcing) during the Anthropocene. The rationale behind discounting the very recent and very ancient ends of a PSMC plot is that relatively few coalescence events are expected to occur during those periods given a population of constant size. The coalescences which PSMC does infer to have occurred during those times therefore have a disproportionate effect on the inferred effective population size, and are thus more likely to reflect error or the fundamental stochasticity of the coalescent process. We note, however, that while only approximately 800 human generations account for the last 20 000 years, 800 generations represent far less time for shorter-lived organisms. Moreover, the expected number of generations to coalescence between any two alleles in a population is proportional to its current effective size, which PSMC estimates based on the overall heterozygosity of the sampled genome. Thus, genomes sampled from less diverse populations should provide greater resolution on the recent past. For example, in the human genome analysed by Li & Durbin [9], approximately 5% of coalescences would be expected (under a constant size model) to occur within the last 20 000 years, given a generation time of 25 years and an *N*_0_ of approximately 8000. Most of the species analysed in the present study are short-lived and rapidly reproducing, giving them short generation times. Reproducible signals of population growth, since the advent of agriculture, are clear for each domesticated host (and each of their symbionts), justifying an important extension of PSMC to the eventful recent past.

Obligate parasites cannot survive except in their hosts, providing a strong *a priori* basis for assuming shared demographic fates. However, even with strong and sustained correlation, alternative causal explanations merit consideration. For instance, climate variation engendering glacial advance and retreat certainly influenced the abundance and distribution of myriad species lacking parasitic or symbiotic dependencies; indeed, leaf-eating primates and pandas appear to have responded, in concert, to shared environmental imperatives [12]. While demographic parallels cannot prove any particular hypothesis to be true, starkly contrasting demographic histories (such as those distinguishing potatoes from the agent of potato blight, prior to potato domestication), can decidedly undermine the case for evolutionary stability in given parasitic relationships.

Our approach fruitfully addresses a long-standing question separating ecology from evolutionary biology: do symbiotic and parasitic relationships long endure? Our demographic approach complements phylogenetic approaches, providing means to examine an interval lasting more than 100 000 years, beginning as early as 1 Ma and continuing as recently as 800 generations ago. Doing so affirmed parallel demographies in three case studies (malaria, *T. spiralis* and a marine symbiosis) and identified a notable exception in the case of potatoes, helping to constrain the origins of potato blight. The rapidly expanding repository of high-quality genome assemblies affords newfound opportunities to consider the longevity of myriad parasitic and symbiotic relationships. Countless other biological dependencies (i.e. plants and their pollinators, predators and their prey, herbivores and their forage, and any pair or group of organisms engaging in synergistic or antagonistic relationships) may be similarly examined with these tools, vastly enriching our understanding of evolution, ecology and epidemiology.

## Supplementary Material

Data details, methodological considerations, and extended analyses

## Data accessibility

Software for implementing this approach, C-PSMC, is described and available for download at https://github.com/lbbhecht/C-PSMC. Novel whole-genome resequencing data are available as SRA (files) at NCBI and can be accessed through BioProject PRJNA489802 with accession numbers SRR7804297-SRR7804300.
