# Supplementary material for: Comparative demography elucidates the longevity of parasitic and symbiotic relationships
Source: Proc Biol Sci. 2018 Oct 3;285(1888):20181032. doi: 10.1098/rspb.2018.1032 (PMC6191686; doi:10.1098/rspb.2018.1032)
Supplement: Data details, methodological considerations, and extended analyses [file rspb20181032supp1.pdf]

Supplemental Information from:

Comparative demography elucidates the longevity of parasitic and symbiotic relationships.

DOI: 10.1098/rspb.2018.1032

Author information

Luke B.B. Hecht[1,2] Peter C. Thompson[1,2], and Benjamin M. Rosenthal[2*]

[1]Oak Ridge Institute for Science and Education, Oak Ridge, TN.

[2]Agricultural Research Service, US Department of Agriculture. 10300 Baltimore Avenue, Beltsville, MD 20705

*to whom correspondence should be addressed Benjamin.Rosenthal@ars.usda.gov

This supplement provides additional details about the samples, subsidiary analyses, and original research results that explore and expand on the capabilities and limitations of comparative historical demography as elaborated in the associated manuscript. We detail more specific findings related to the four biological systems studied here (malaria*, Trichinella spiralis*, *Symbiodinium minutum*, and potato blight systems), emphasizing themes such as replicability, specificity of a parasite's fit to a particular host species, geographic specificity of host-parasite demographic parallels, and the applicability of PSMC to organisms (such as potatoes and most other crops) where successive genome duplications and uncertain genome assemblies may inflate estimates of heterozygosity and thereby bias estimates of ancient population size.

Sample Information

Supplemental Table S1: Host and symbiont species, sample abbreviations, and the form in which the sample was obtained. The *P. falciparum* isolates marked with asterisks were assembled as pseudodiploids from multiple sets of published reads, as described below.

Sample abbreviation	Species	Specimen form
Human	*Homo sapiens*	Published PSMC
Gorilla	*Gorilla beringei*	Published PSMC
Sus-EUboar	*Sus scrofa* (wild boar from Europe)	Published reads
Sus-EUpig	*Sus scrofa* (domestic pig from Europe)	Published reads
Sus-CNboar	*Sus scrofa* (wild boar from China)	Published reads
Sus-CNpig	*Sus scrofa* (domestic pig from China)	Published reads
Aiptasia	*Aiptasia pallida*	Published reads
Acropora	*Acropora digitifera*	Published reads
Potato	*Solanum tuberosum*	Published reads
Agamb	*Anopheles gambiae*	Published reads
Pfalc-AS1	*Plasmodium falciparum* (from Cambodia)	Published reads*
Pfalc-AS2	*Plasmodium falciparum* (from Cambodia)	Published reads*
Tspir-US1	*Trichinella spiralis* (from USA)	Biological sample
Tspir-US2	*Trichinella spiralis* (from USA)	Biological sample
Tspir-CN1	*Trichinella spiralis* (from China)	Biological sample
Tspir-CN2	*Trichinella spiralis* (from China)	Biological sample
Smin	*Symbiodinium minutum*	Published reads
Pinf	*Phytophthora infestans*	Published reads

Supplemental Table S2: Summary of files used for genome assembly and heterozygote base-calling prior to PSMC analyses.

**Species**	**Reference Accession #**	**Isolate**	**SRA**	**Mean Sequencing Depth**	**% Ref-seq coverage**
Plasmodium falciparum	GCA_000002765.1	AnlongVeng	SRR2318686	94	99.9
			SRR2318688		
		KampongLpov	SRR2318021	40	96.2
			SRR2318061		
Anopheles gambiae	GCF_000005575.2AgamP3_genomic	PEST	ERR501422	13	95.2
Homo sapiens	HGDP00521	Human-French	PSMC from original publication*		
Gorilla gorilla			PSMC from original publication*		

Pan troglodytes			PSMC from original publication*		
Trichinella spiralis	GCA_000181795.2	China-ISS534	SRR7804297	35	90.3
		China-TY2	SRR7804300	17	89.2
		Western-NA-Shisler1	SRR7804298	18	88.1
		Western-NA-G279	SRR7804299	24	88.3
Sus scrofa	GCA_000003025.6	European-WBNL	PSMC from original publication*		
		European-WBIT	PSMC from original publication*		
		North Chinese-WBNC	PSMC from original publication*		
		South Chinese-WBSC	PSMC from original publication*		
Aiptasia pallida	GCA_001417965.1	CC7	SRR1648348	85	85.0
Acropora digitafera	GCA_000222465.2_Adig_1.1_genomic		DRR001405	16	86.6
			DRR001406		
			DRR001407		
			DRR001408		
			DRR001409		
			DRR001410		
			DRR001411		
			DRR001412		
			DRR001413		
			DRR001414		
			DRR001415		
Symbiodinium minutum	GCA_000507305.1_ASM50730v1_genomic	Oku, Okinawa, Japan	DRR003842	114	99.6
			DRR003843		
			DRR003844		
			DRR003845		
			DRR003848		
			DRR003849		
			DRR003850		
			DRR003851		
			DRR003854		
			DRR003859		
			DRR003862		
Solanum tuberosum	GCA_000226075.1_SolTub_3.0_genomic	DM 1-3 516 R44	SRR307623	34	94.2
			SRR307648		

			SRR307666		
			SRR307667		
			SRR307668		
			SRR307675		
			SRR307677		
			SRR307682		
Phytophthora infestans	GCA_000142945.1_ASM14294v1_genomic	T30-4	ERR019607	6.9	87.0
			ERR019608		
			ERR019609		
			ERR019610		
			ERR019611		
			ERR019612		
			ERR019613		
			ERR019614		
			ERR019615		
			ERR019616		
			ERR019632		
			ERR019633		
			ERR019634		

* Original PSMC plot data were generously provided by authors of previous publications[8,10]

Figures referred to in Methods:

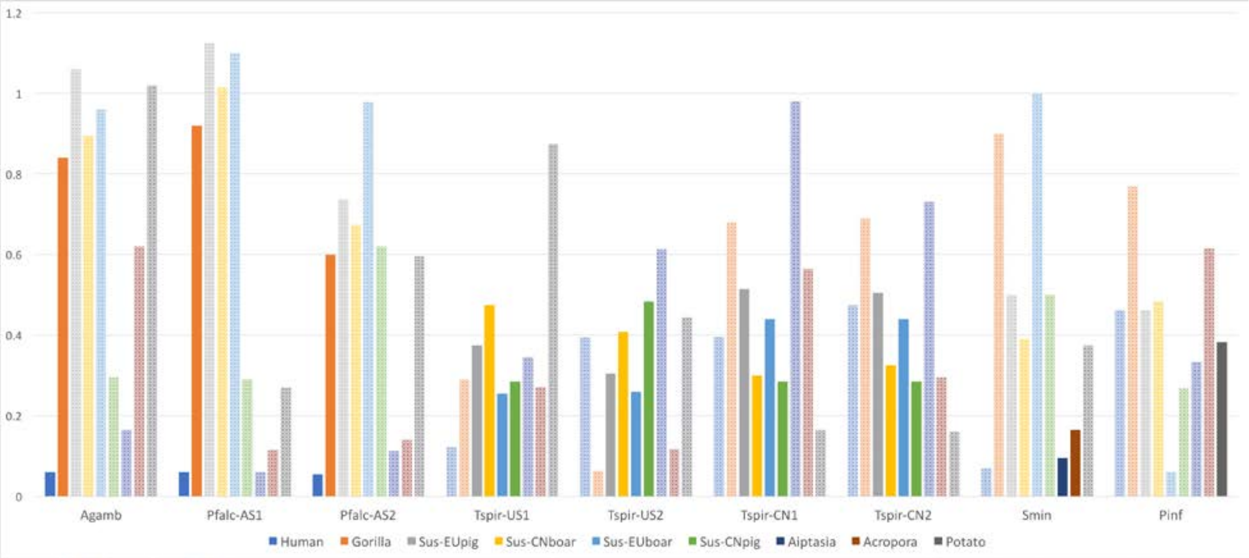

Figure S1. Comparison of minimum average slope difference for all considered host-symbiont pairs. Bars representing the fit of biologically plausible hosts are highlighted, while apocryphal matches are faded, but shown for context. In all cases, the true host is preferred from the arena of plausible hosts (e.g. European boar > Asian boar for European *T. spiralis*), but in some cases an apocryphal match scores better (e.g. European boar > potato for *P. infestans*).

Supplemental Table 3: Range of plausible generation times (in years) assumed for each symbiont genus.

**Species**	**Generation time (yr)**
A. gambiae	0.01 - 0.12
P. falciparum	0.3 - 0.8
T. spiralis	0.8 - 5.0
S. minutum	0.0 - 1.0
P. infestans	0.0 - 0.3

Effect of variant-calling methodology on PSMC

We tested the sensitivity of PSMC to alternative variant-calling methods, which may influence the number and distribution of heterozygotic base calls in the consensus sequence. We observed little difference in shape or scaling between PSMC plots of *T. spiralis* isolates based on heterozygote-calling performed, alternatively, by Geneious or the mpileup function of Samtools (samtools mpileup -uf Genome.fasta test.sorted.bam | bcftools view -cg - | vcfutils.pl vcf2fq > consensus.fq) (Supplemental Figure S2).

The heterozygote frequencies found in the PSMC input files (.psmcfa) generated from these consensus fasta files were also similar irrespective of the variant-calling program, with the Geneious consensuses generally having a greater proportion of missing data, suggesting the filtering thresholds we applied (75% consensus, >5x coverage) were slightly more stringent than the Samtools defaults (Supplemental Table 4). Nadachowska-Brzyska et al. (2016) [1] reported that increasing their per-site filtering thresholds shifted their PSMC curve toward the present, and this effect is also apparent to a minor degree in our plots (Supplemental Fig. S2). The same authors found that more stringent filtering helped to resolve population fluctuations in PSMC plots, though the significance of this faded for average read depths greater than ~18x.

Supplementary Table 4: Estimated number of Heterozygotes (Hets)/Homozygotes (Homs)/Indeterminate (Ind) positions (x100,000) called by Samtools and Geneious in four isolates of *T. spiralis.*

***T. spiralis* isolate**	**Samtools** (Hets/Homs/Ind)	**Geneious** (Hets/Homs/Ind)
Western-NA-Shisler1	50/407/72	43/401/85
Western-NA-g279	39/417/72	28/418/82
China-ISS534	216/259/54	170/308/50
China-TY2	149/315/65	127/323/79

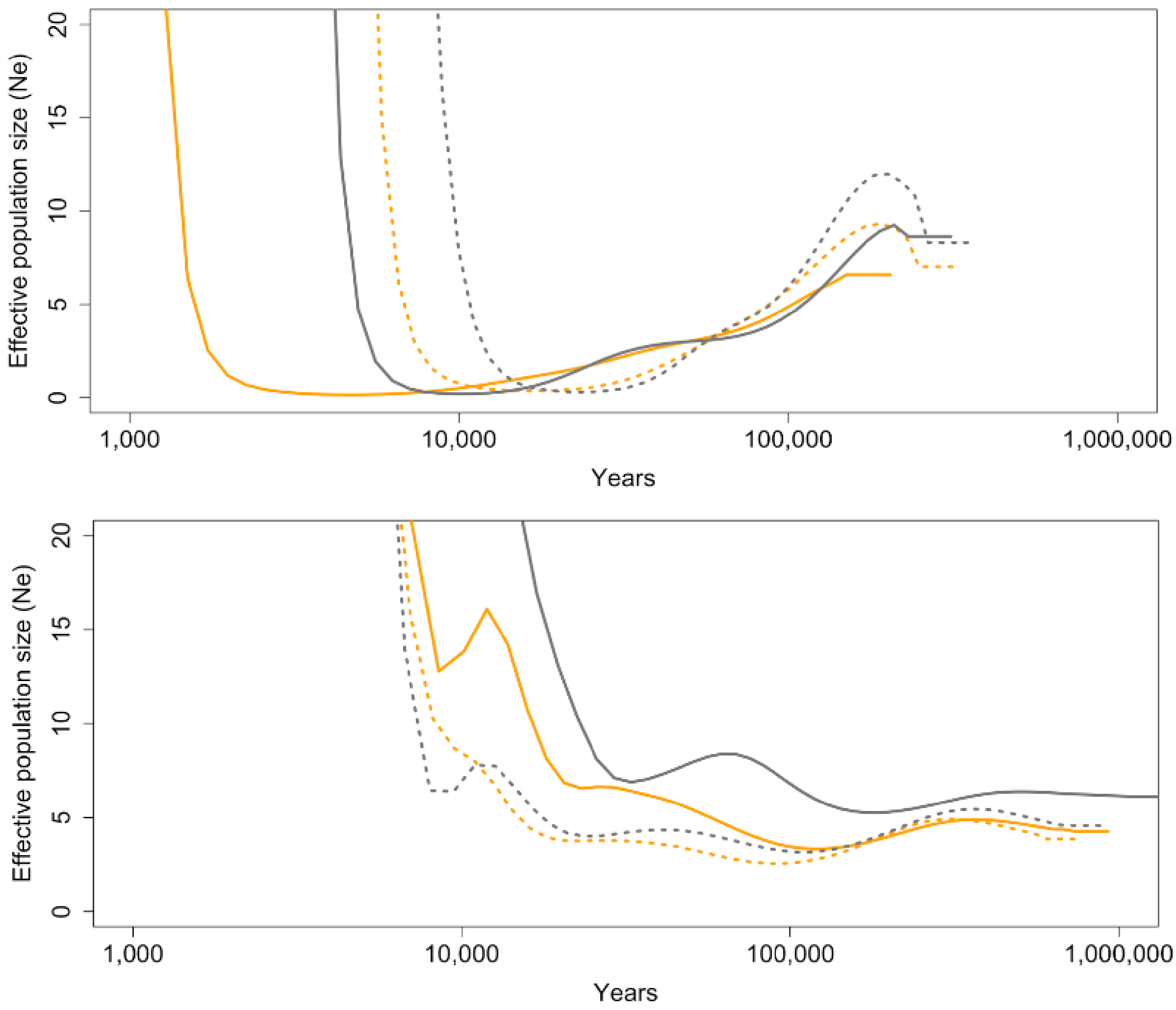

Figure S2. PSMC plots based on consensus sequences generated by Geneious (orange) and Samtools (grey), for two *T. spiralis* isolates from America (left) and two from China (right)

In general, joint histories of species pairs could be traced from about 500,000 years ago (where years result from joint estimates of mutation rate and generation time) until a few thousand years ago. In considering the evolutionary longevity of particular parasitic or symbiotic relationships, our methods appear well-suited to that interval (and no longer). It was often the case that one, but not both, genomes in a given pair provided temporally deeper inferences on demographic history.

Malaria system

The origin and expansion of the agent of malaria, *P. falciparum*, is tied to human demographic history [2,3]. The most recent evidence indicates that *P. falciparum* shifted from gorillas to humans between 60,000 and 130,000 years ago [4], though chimpanzees were previously suspected as the proximate source [5,6]. When humans began clearing land for agriculture, anopheline mosquito numbers increased accordingly, resulting in increased malaria transmission and presumably increased *P. falciparum* population sizes. Therefore, we sought to

examine the demographic changes written in the genomes of hosts, vectors, and parasites to confirm our understanding of falciparum malaria in humans.

Plasmodium falciparum is only briefly diploid in its anopheline mosquito definitive hosts, and to our knowledge has not been sequenced in that life-history stage. Therefore, we mapped the distribution of potentially heterozygous positions by creating “pseudodiploid” genomes in silico by mapping short reads from two haploid sequencing projects derived from *P. falciparum* isolated in the same geographic region to the *P. falciparum* 3D7 chromosomes (the most complete representation of the *P. falciparum* genome to date). We took these assemblies to represent the joining of haploid gametes into transient diploid ookinetes. We selected sequencing projects with approximately equivalent numbers of reads in order that heterozygous bases could be determined based on read depths as above, assuming comparable coverage of chromosomes by each deeply sequenced genome. The resulting consensus chromosomes were used for downstream analysis, including estimates of relative generation time (Supplemental Figure S3) that well match empirical estimates based on clinical and entomological observation. Producing pseudodiploids in this way, we note, could render any haploid organisms amenable to PSMC analysis.

Plasmodium falciparum notably parallels the bottleneck evident for human beings and their subsequent ascendency in the last ten thousand years; by comparison, other great apes have experienced stable or declining effective population sizes since the Neolithic (Supplemental Figure S4). Similar demographic patterns have been inferred for primates circa 100 kya-1 Mya, and would be expected to eventually converge on the shared demography of our common ancestors; subsequent to divergence, it would not be surprising to find that various primate species were subjected to common beneficial and injurious environmental conditions.

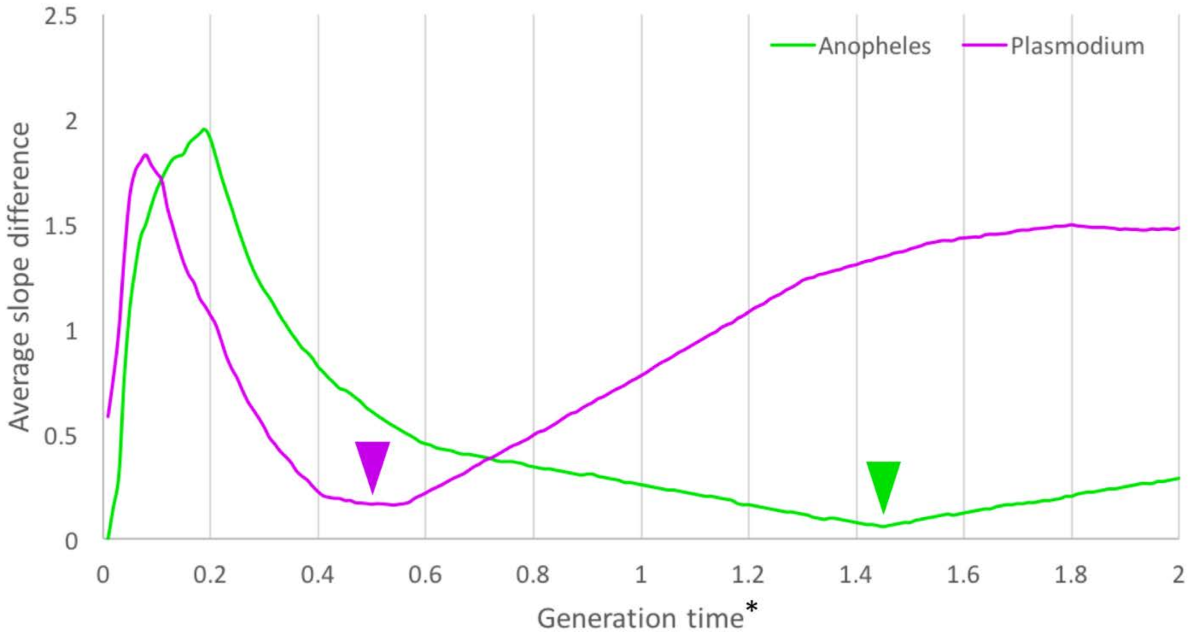

Figure S3. PSMC curve-fit to French Human as a function of generation time for *A.gambiae* (green) and *P. falciparum* (purple), assuming a generation time of 25 years for humans and identical per-generation mutation rates of $2.5e^{-8}$. The lowest average slope difference was found

at 1.43 years/generation for *A.gambiae* and 0.48 years/generation (~23 weeks/generation) for *P. falciparum*. However, the slower *Drosophila* mutation rate ($1.1e^{-9}$) may be more appropriate for mosquitoes [7], which would imply an *A. gambiae* generation time of 0.057 years, or approximately one generation every 3 weeks, in line with empirical estimates based on knowledge of mosquito ecology.

Chimpanzees had previously been considered the penultimate host for the ancestors of falciparum malaria until more closely related parasites were discovered in gorillas. Because ancestors of chimpanzees may have served as earlier hosts to ancestors to falciparum malaria, we considered the demographic history of *P. falciparum* in relation to that published by Prado-Martinez et al. (2013) [8] for various populations of chimpanzees (*P. troglodytes ellioti*, *P.t. schweinfurthii*, *P.t. troglodytes*, *P.t. verus*). While all Old World primates (including *H. sapiens*) share certain features of demographic history, the histories of various sub-populations of chimpanzees notably differ (Supplemental Figure S4). The demographic history of *P. falciparum* tracks human demography best, especially during the most recent historical interval, when human populations grew exponentially while other great apes did not. However, of the considered chimpanzee populations, *P.t. schweinfurthii* was the most consistent with human and malaria demography in the range of 80K – 1M years ago.

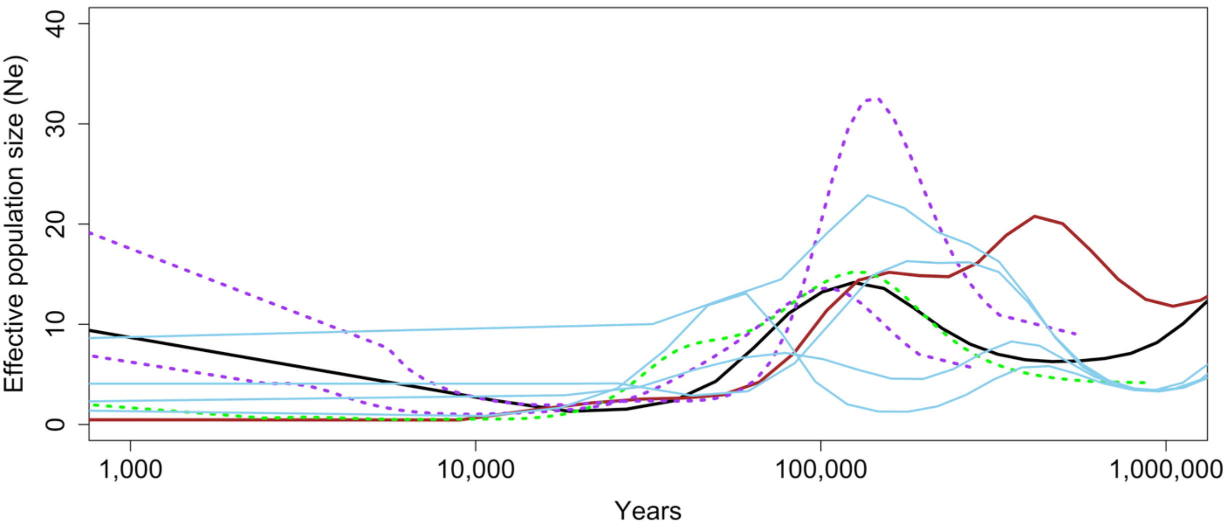

Figure S4. Estimated demographic histories for *P. falciparum falciparum* (violet) and *A. gambiae gambiae* (green) in relation to that for human (black) and gorilla (brown) and multiple subspecies of chimpanzee (blue). Gorilla populations grew and shrank in parallel with humans, Anophelines, and *P. falciparum*, with steep declines from 200,000 ybp to 70,000 ybp, followed by a small stable population between 70,000 and 11,000 ybp. From ~100-11 kya, gorilla populations declined in concert; prior to then, their populations were notably larger and since then notably smaller. *P. falciparum* and mosquito closely parallel human demography for a

considerable period, including growth in the most recent past unique, among primates, to human populations. Recent growth in the mosquito population, while present, is much less dramatic than that observed in the human and parasite populations. Note that human and gorilla effective population sizes were uniformly scaled up by a factor of 10 to be more easily visible in this figure.

***Trichinella spiralis* system**

Trichinella spiralis is contracted by the consumption of meat in which larval parasites have encysted. Adult pairs mate within weeks, and disseminate new larvae to the tissues whereupon the next generation of larvae establish chronic infections that endure for the life of the host. Thus, infections are acquired after weaning and are transmitted at death. The mean generation time of the parasite would be shortened to the extent that infection reduced swine longevity and to the extent that the parasite also exploits shorter-lived hosts, such as rats. The curve-fitting procedures described above suggested ~ 4 parasite generations per wild boar generation (or 1.1 years, assuming 5-year generations for wild boar).

The inferred relative generation time of *T. spiralis* was inconsistent in Asia and in Europe (Supplemental Figure S5), probably owing to limited complexity and depth in the demographic history reconstructions of European *T. spiralis*. Since domestication, pigs have undergone a population explosion, and our analysis illustrate commensurate exponential growth in the size of the parasite population (Supplemental Figure S6). Wild boar have interbred with domesticated pigs, leaving tracts of homozygosity in wild boar genomes [9] that may inflate estimates of recent population growth in wild boar.

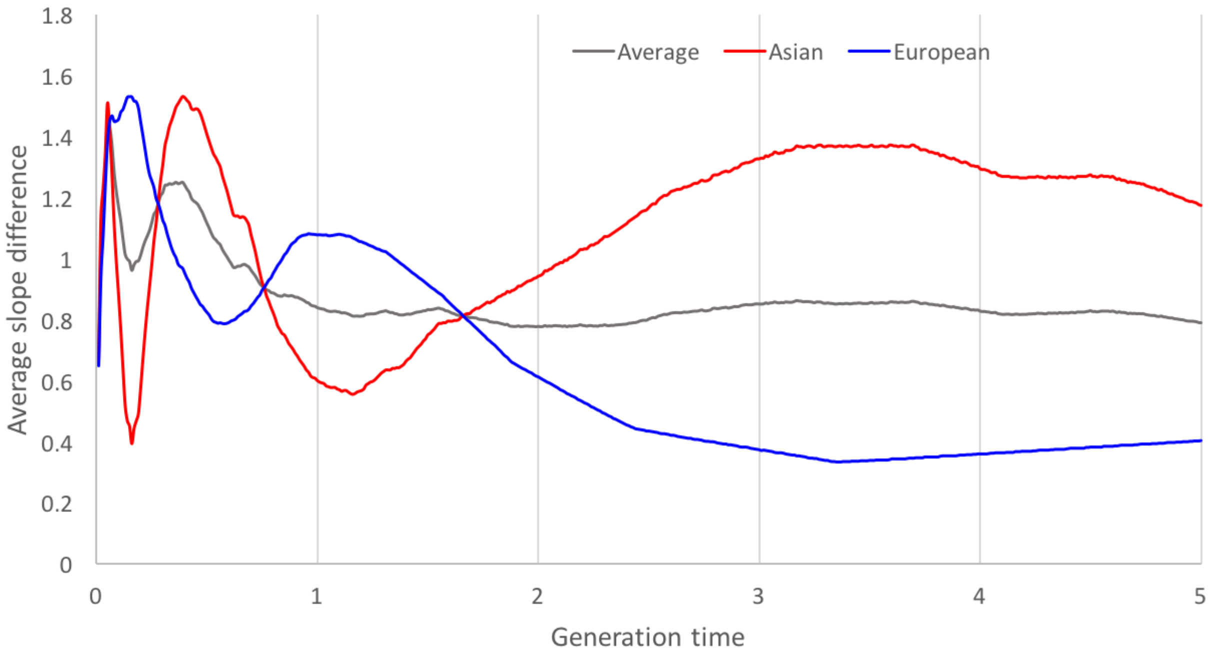

Figure S5. Fit of European (blue) and Chinese (red) *T. spiralis* to sympatric *S. scrofa* as a function of their generation time. Given 5 years per boar generation, the lowest average slope difference between the average of the two regional fits (grey line) stabilizes at approximately 1.1

years per *T. spiralis* generation, which is the regional optimum for the Asian *T. spiralis*. Because the Asian genomes record a deeper and more complex demographic history than the European ones, we elected to proceed with 1.1 years/generation as the species optimum generation time for *T. spiralis*. Note that although the Asian *T. spiralis* slope difference is minimized at <6 weeks, this biologically implausible result derives from few overlapping time intervals (between parasite and host).

As stated, the demographic plots for western *T. spiralis* were generally lacking in complexity, depriving curve-fitting procedures of information needed to achieve precise estimates (Supplemental Figure S5). Nonetheless, their optimal fit is consistent with a decline in pig populations in both Europe and Asia that has previously been interpreted as having been caused by the LGM [10]. The inferred histories of Asian *T. spiralis* reach deeper in time and show greater demographic variability over time. Most notably, in contrast to European *T. spiralis* and to both European and Asian *S. scrofa*, the effective population size of Asian *T. spiralis* does not decline over the last ~50,000 years, but stabilizes and grows. All pigs and *T. spiralis* eventually gave way to dramatic growth over the Holocene.

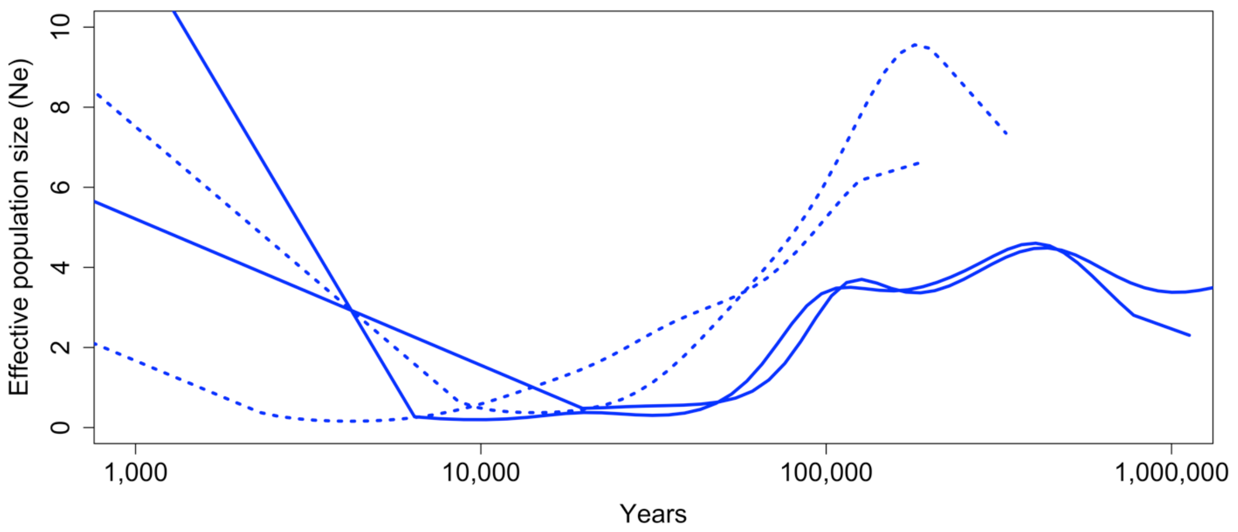

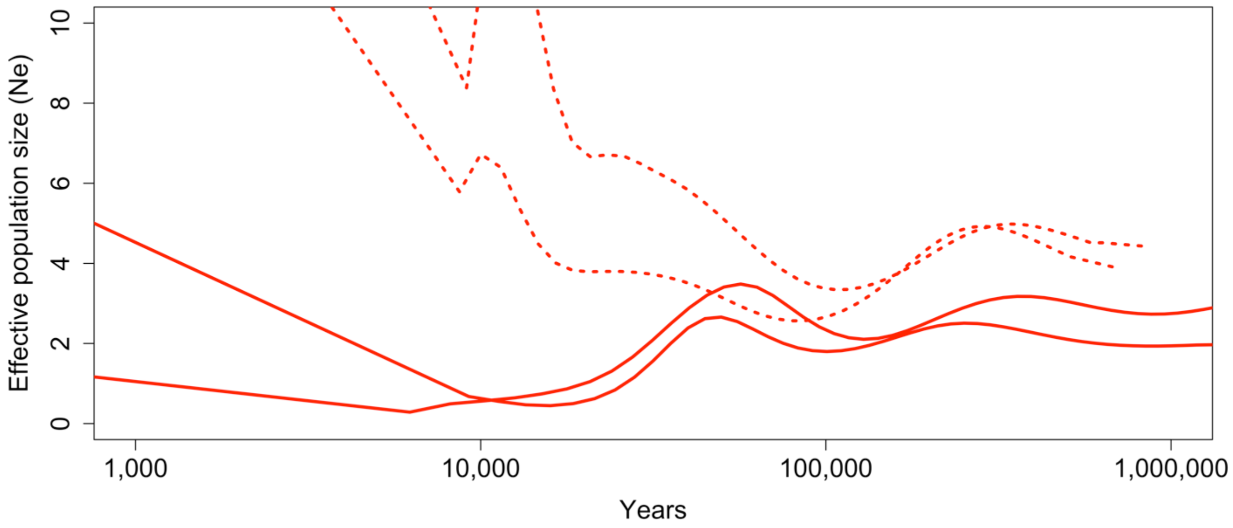

Figure S6. Regionally-specific reconstruction of *T. spiralis* demography in relation to sympatric wild boar. Regional distinctions in the histories of wild boar are recapitulated in the histories of the parasite prior to the age of swine domestication. Since domestication, parasite populations in each region have undergone precipitous growth. The PSMC curves are those reconstructed for wild boar by Groenen et al, 2012) [10].

***Symbiodinium* system**

A long-term demographic association between the anemone *Aiptasia pallida* and its symbiont *Symbiodinium minutum* might not have been expected owing to expulsion and die-offs of symbionts during periods of environmental stress [11]. Cnidarians, including anemones and corals, sometimes acquire new species after heat-induced bleaching [11]. Although such associations might therefore prove ephemeral, the population of *S. minimum* tracked that of *A. pallida* remarkably closely (Supplemental Figure S8). The demographic histories of all three species were similar until approximately 10-20 kya, following the LGM, when the coral population began a period of extreme growth while the anemone and symbiont populations began to decline.

We employed equivalent curve-fitting procedures so as to determine the best-possible demographic match over time, and derived a relative generation time of ~0.5 against the actual host, *Aiptasia pallida*, and 0.97 against the apocryphal host, *Acropora digitifera* (Supplemental Figure S8). Notably, the estimated slope difference between host and symbiont demographic curves was less for the natural pair at almost every considered value of relative generation time (Supplemental Figure S7); the average slope difference between *S. minutum* and that of its actual host (~.09) was notably less than to that of its apocryphal host (~0.17) when optimally scaled to each. Supplemental Figure S8 depicts the histories of all three species, assuming a relative generation time of 0.52 for *S. minutum* (fit to *Aiptasia pallida*).

Their shared history in the Red Sea may partially explain the evidently shared demographic histories of the anemone and its symbiont (in contrast to that of the coral, obtained from Okinawa in the East China Sea). Although oceanic coral systems generally prosper when the global climate cools, glacier formation during the last glacial maximum resulted in a 120m drop in sea level, halving the surface area of the Red Sea, significantly reducing exchange with the Indian Ocean through the Strait of Bab el Mandab, and increasing the sea's salinity by about 50% [12,13]. These events would likely have reduced the census size of the anemone population, as well as fragmenting it within the Red Sea and between the sea and ocean, further reducing the effective population size of the anemone. Smaller, fragmented host populations would likely have constrained growth of the symbiont. Subsequent rising seas may have engendered population growth of the anemone and its algal symbiont owing to increased habitat availability and increased gene flow among once-separated populations (Supplemental Figure S8).

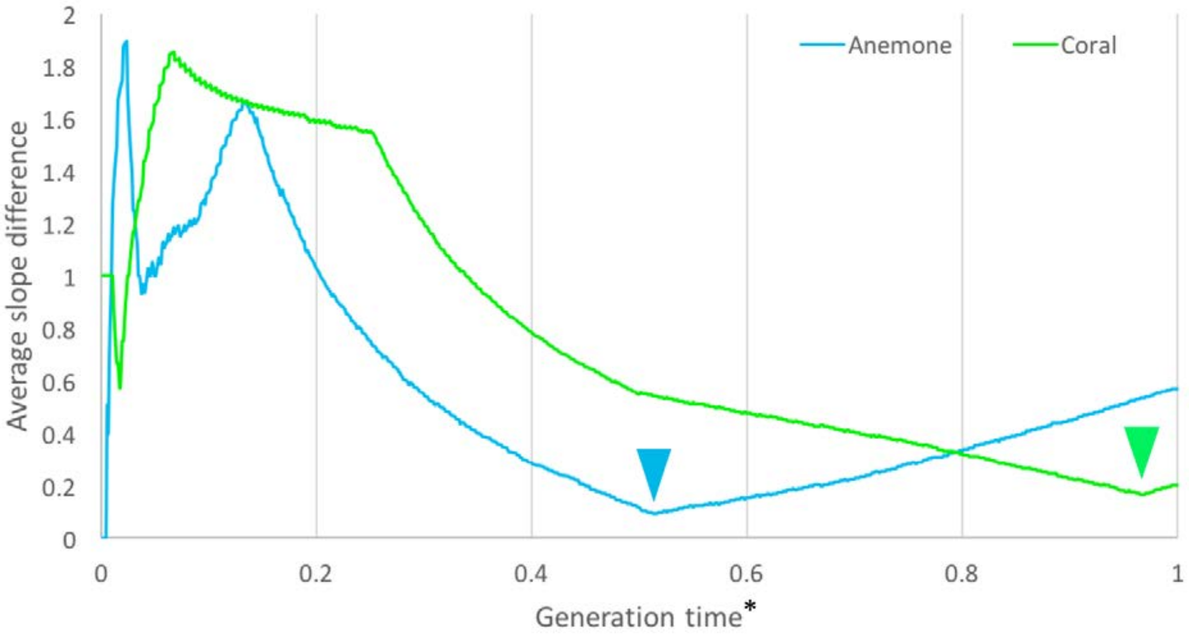

Figure S7. The slope difference between *S. minutum* and its natural host (*Aiptasia pallida;* blue line) was less than to another marine invertebrate, the coral *Acropora digitifera* (green line). The fit to *Aiptasia pallida* was optimized at a relative generation time of approximately half (0.52x) that of the host. Optimal fitting to *Acropora digitifera* would require almost identical generation times for host and symbiont (0.97x).

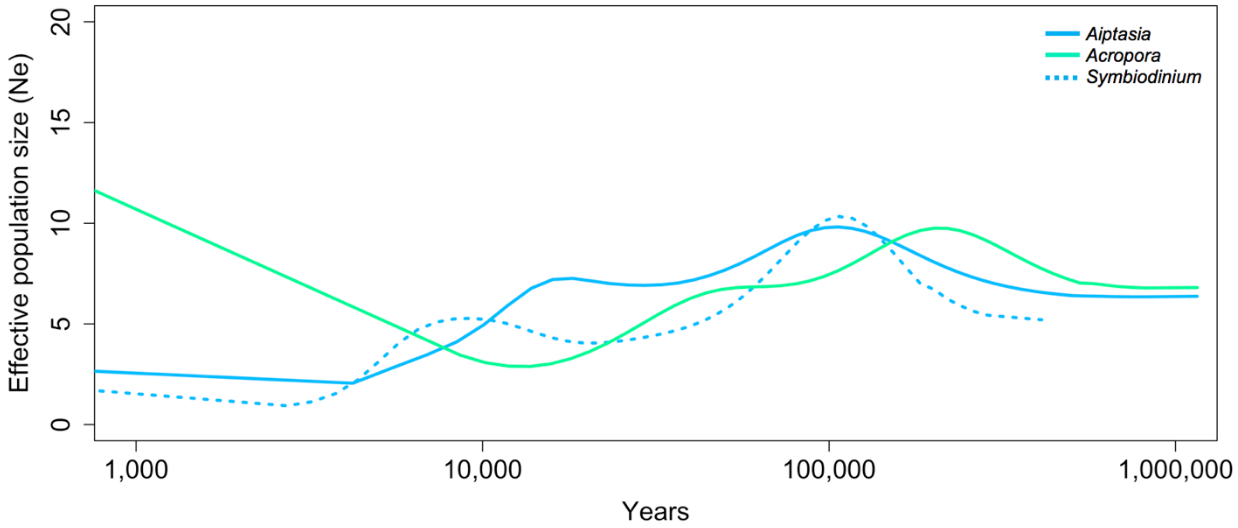

Figure S8. Demographic histories of coral (*Acropora digitifera*), anemone (*Aiptasia pallida*), and the anemone's symbiont (*S. minutum*), assuming annual generations for the coral and anemone and biannual generations for the symbiont. Rises and falls of *S. minutum* (dotted blue line) approximate those for its extant host, *Aiptasia pallida* (solid blue line) for approximately 1 million years. A similar pattern of growth and decline is observed, albeit out of sync, for *Acropora digitifera*, but the demographic trajectory of *Acropora digitifera* separates in the last 20,000 years, experiencing rapid population growth while the anemone and its symbiont decline and stabilize.

Potato system

Infections of potatoes (*Solanum tuberosum*) with the potato blight *Phytophthora infestans* caused one of the greatest human disasters in agricultural history, resulting in the death or emigration of 20% of the Irish in the mid-1800's. Potatoes originated in the Andes and may have been domesticated twice. Biogeographic data suggest the central Mexican highlands as the ancestral home of *P. infestans,* achieving global reach only after the potato domestication in the Neolithic and the globalization of agriculture beginning 500 years ago. *P. infestans* appears restricted to tuber-forming species of Solanum and does not easily cross with other species of *Phytophthora*. The closest known relatives to *P. infestans* (*P. andina, P. ipomoea, P. mirabilis,* and *P. phaseoli*) are also pathogens of plants native to the Neotropical highlands. However, other closely related congeners (*P. iranica, P. clandestina, P. tentacultate*) afflict diverse plant types (eggplant, clover, chrysanthemum) in diverse regions (Asia, Europe); an even greater diversity of host types and biogeographic regions characterize more inclusive groups of *Phytophthora,* including species infecting orchids native to Indonesia, lilacs in the Balkans, rhododendron native to the Himalayas, raspberry native to Northern Europe and Asia, and Douglas fir in Western North America. This broader evidence for host switching provides valuable context to our results, suggesting parallel demography only during the most recent past.

Prior to potato domestication and cultivation, potatoes and the agent of potato blight shared no obvious episodes of concerted population growth or contraction. Lacking such compelling information, and with a relatively short and simple reconstructed history of *P. infestans* to overlap, our curve-fitting algorithm easily identified a clear preferred relative generation time for

the two at ~0.14 *P. infestans* generations per potato generation (which was assumed to be one year) (Supplemental Figure S9). Adopting this temporal scalar resulted in an average slope difference (0.38 on a scale of 0-2) that was as poor as the best fit between apocryphal species pairs (such as wild boar), even with relatively tight constraints on plausible generation times for the pathogen (<3 months [14]). Among poor alternatives, the best-fit curves register by reference only to the recent (post-domestication) interval of population growth in potatoes. This is preceded by ~20,000 years of decline in potatoes and relative stasis in *P. infestans* (Supplemental Figure S10). These findings are consistent with a close affinity of *P. infestans* with potatoes only since their domestication.

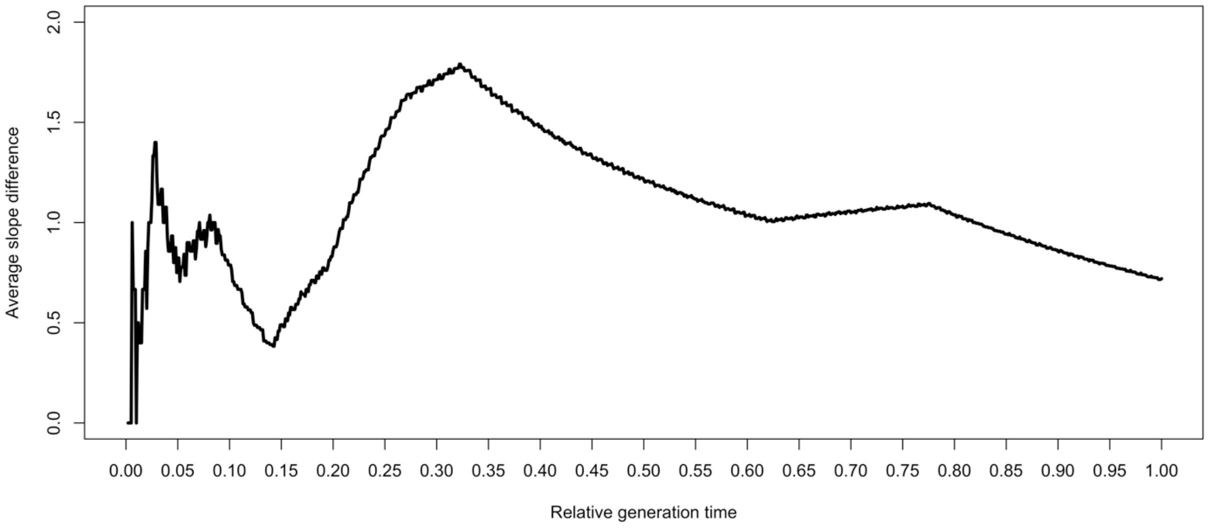

Figure S9. Fit of *P. infestans* demographic history to that of potatoes as a function of relative generation time. The optimum is reached when a single potato generation equals ~10 generations of *P. infestans.* This short generation time is based on concerted growth in the host and pathogen only since the domestication of potatoes in the last ~8,000 years (Figure S10), suggesting that shared demography may have been limited to this recent temporal interval.

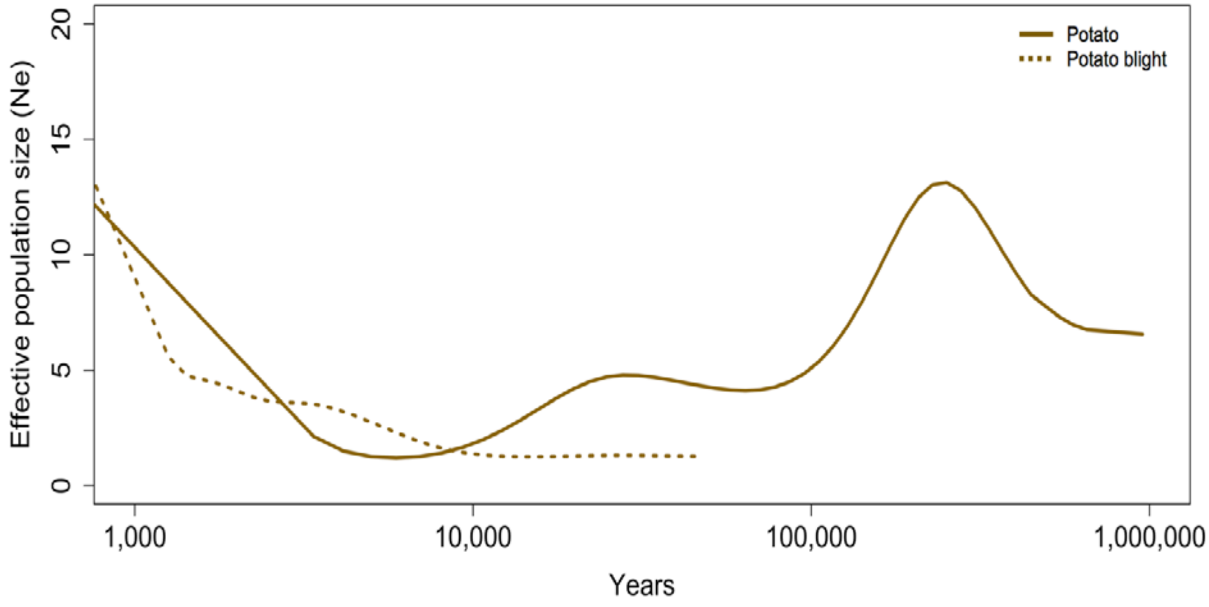

Figure S10. Contrasting demographic histories of potato and *P. infestans* the agent of potato blight excepting since domestication. The only demographic feature indicating shared history in this plant/pathogen system relates to recent growth in each. Here, the demographic history of potato (solid line) is depicted against the history of the fungal pathogen assuming the optimal relative generation time, which does not convincingly match the history of potatoes prior to potato domestication.

Noting an ancient tripling of chromosomal content in dicot plants [15], we were careful to assess whether, how, and to what extent demographic plots might be biased if reads derived from paralogs in the potato genome were mismapped to the same position, thereby inflating estimates of heterozygosity. We examined this potential source of bias by filtering regions of the potato genome sequenced to especially high coverage (arguably over-represented owing to their occurrence as multiple copies). Prior to filtering, the potato genome dataset had an average sequencing depth of 31.8 reads per site with a large standard deviation (148.5). After filtering, the mean sequencing depth and standard deviation was reduced to 12.4 and 13.3, respectively. Filtering resulted in removal of approximately 1/5 of each scaffold for consideration by PSMC. After filtering, about 40 million bases of contiguous sequence in 10 scaffolds were available for analysis. Filtering high coverage areas reduced the overall heterozygosity in the potato genome from 1.19% to 0.895%. It also reduced the length of the scaffolds used for secondary PSMC analysis from 43,353,824 bp to 36,083,464 bp (17% removed from consideration). Confirming simulations entertained by Li and Durbin (2011) [16], removing suspected paralogous regions did not affect the overall shape of curve, but did reduce the estimate of the most ancient populations (Supplemental Figure S11). The essential demographic signal to remain stable, further substantiating the conclusion that potatoes and the fungal agent of potato blight did not experience parallel demographic histories until the age of potato domestication, beginning ~9,000 years ago.

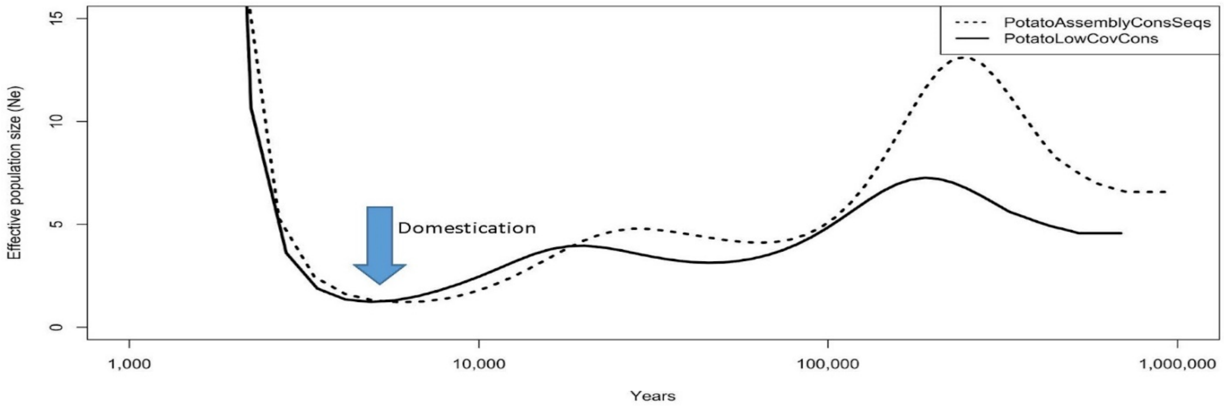

Figure S11. Effect of removing suspected paralogs from the potato genome prior to estimating its demographic history using PSMC. The PSMC plot derived from the potato genome assembly (dotted line) was compared to an equivalent analysis performed on a dataset from which anomalously deeply sequenced regions were removed (solid line), owing to the suspicion that paralogous gene copies derived from genome duplications might be inflating estimates of heterozygosity. As predicted (Li and Durbin, 2011) [16], removing these sequences did reduce estimates of ancient population size. However, this quality control step did not fundamentally

alter the inference as to when ancestral potato populations grew or contracted, and specifically had no appreciable effect on demographic estimates within the last 30,000 years.

1. Nadachowska-Brzyska K, Burri R, Smeds L, Ellegren H. 2016 PSMC analysis of effective population sizes in molecular ecology and its application to black-and-white Ficedula flycatchers. *Mol. Ecol.* **25**, 1058–1072. (doi:10.1111/mec.13540)

2. Rich SM, Licht MC, Hudson RR, Ayala FJ. 1998 Malaria's Eve: Evidence of a recent population bottleneck throughout the world populations of *Plasmodium falciparum*. *Proc. Natl. Acad. Sci.* **95**, 4425–4430.

3. Volkman SK *et al.* 2001 Recent Origin of *Plasmodium falciparum* from a Single Progenitor. *Science* **293**, 482–484. (doi:10.1126/science.1059878)

4. Liu W *et al.* 2010 Origin of the human malaria parasite *Plasmodium falciparum* in gorillas. *Nature* **467**, 420–425. (doi:10.1038/nature09442)

5. Escalante AA, Ayala FJ. 1994 Phylogeny of the malarial genus *Plasmodium*, derived from rRNA gene sequences. *Proc. Natl. Acad. Sci.* **91**, 11373–11377.

6. Rich SM *et al.* 2009 The origin of malignant malaria. *Proc. Natl. Acad. Sci.* **106**, 14902–14907. (doi:10.1073/pnas.0907740106)

7. O'Loughlin SM, Magesa SM, Mbogo C, Mosha F, Midega J, Burt A. 2016 Genomic signatures of population decline in the malaria mosquito *Anopheles gambiae*. *Malar. J.* **15**, 182. (doi:10.1186/s12936-016-1214-9)

8. Prado-Martinez J *et al.* 2013 Great ape genetic diversity and population history. *Nature* **499**, 471–475. (doi:10.1038/nature12228)

9. Manunza A *et al.* 2016 Romanian wild boars and Mangalitza pigs have a European ancestry and harbour genetic signatures compatible with past population bottlenecks. *Sci. Rep.* **6**, 29913. (doi:10.1038/srep29913)

10. Groenen MAM *et al.* 2012 Analyses of pig genomes provide insight into porcine demography and evolution. *Nature* **491**, 393–398. (doi:10.1038/nature11622)

11. Baker AC. 2003 Flexibility and specificity in coral-algal symbiosis: diversity, ecology, and biogeography of *Symbiodinium*. *Annu. Rev. Ecol. Evol. Syst.* **34**, 661–689. (doi:10.1146/annurev.ecolsys.34.011802.132417)

12. Rohling EJ. 1994 Glacial conditions in the Red Sea. *Paleoceanography* **9**, 653–660. (doi:10.1029/94PA01648)

13. Biton E, Gildor H, Peltier WR. 2008 Red Sea during the Last Glacial Maximum: implications for sea level reconstruction. *Paleoceanography* **23**, PA1214. (doi:10.1029/2007PA001431)

14. Goodwin SB. 1997 The population genetics of *Phytophthora*. *Phytopathology* **87**, 462–473. (doi:10.1094/PHYTO.1997.87.4.462)

15. Jaillon O *et al.* 2007 The grapevine genome sequence suggests ancestral hexaploidization in major angiosperm phyla. *Nature* **449**, 463–467. (doi:10.1038/nature06148)

16. Li H, Durbin R. 2011 Inference of human population history from individual whole-genome sequences. *Nature* **475**, 493–496. (doi:10.1038/nature10231)
